# The Use of Specialized Neuropsychological Training for the Rehabilitation of Patients with Neglect Syndrome

**DOI:** 10.11621/pir.2025.0201

**Published:** 2025-05-01

**Authors:** Maria S. Kovyazina, Nataliya A. Varako, Georgiy K. Stepanov, Daria D. Yurina, Victoria A. Propustina, Svetlana A. Vasilyeva, Vadim D. Daminov, Yury P. Zinchenko

**Affiliations:** a Lomonosov Moscow State University, Russia; b Research Center of Neurology, Moscow, Russia; c Federal Scientific Center of Psychological and Multidisciplinary Research, Moscow, Russia

**Keywords:** neuropsychology, rehabilitation, stroke, neglect syndrome, visuospatial search, training

## Abstract

**Background:**

Currently, insufficient attention is being paid to methods of rehabilitation of patients with neglect syndrome. It is necessary to create an effective way to overcome this condition.

**Objective:**

After we developed and tested a specialized rehabilitation training program for patients with neglect syndrome, we sought to verify that the designed program would have a positive impact on the indicators of visuospatial search in these patients, in contrast to a control group. The patients underwent the specialized training, and improvements in the indicators of visuospatial search were observed, which minimized manifestations of left neglect.

**Design:**

The study consisted of two stages: 1) the development and approbation of the author’s task aimed at diagnosing visuospatial neglect syndrome, and 2) the development and approbation of the specialized rehabilitation training and evaluation of its impact on the indicators of visuospatial search in patients with neglect syndrome. Eighty-three patients with lesions of the right hemisphere of the brain took part in the first stage of the study; 37 patients with left-sided visuospatial neglect syndrome took part in the second.

**Results:**

The results of the ANOVARM data analysis indicated statistically significant changes in the visuospatial search of the experimental group’s patients. Specifically, there was an increase in the number of head turns to the left (F = 18.127, p<.05) and a decrease in the proportion of missed stimuli on the left (F = 18.367, p<.05). Additionally, the total time to complete the tasks presented by the author’s methodology increased after participation in the specialized rehabilitation training. An increase in this indicator may suggest that patients in the experimental group were becoming more aware of the search process. There were no significant differences in the indicators of visuospatial search between patients in the two control groups.

**Conclusion:**

After completing a specialized rehabilitation program to overcome left-sided neglect, patients with neglect syndrome demonstrated improved visuospatial search indicators and increased overall visual activity. The training also had non-specific effects, such as increased motivation, more engagement in the rehabilitation process, and more willingness to participate in other individual and group classes.

## Introduction

Neglect syndrome is a condition in which patients are unable to detect, respond to, or navigate stimuli located on the contralateral side of the affected hemisphere of the brain (Heilman, Valenstein, & Watson, 2000). Unilateral spatial neglect is a syndrome that can make patients dependent on others due to the difficulties and dangers they face. In the brain injury clinic, the presence of neglect usually correlates with unfavorable outcomes regarding functioning after injury; in particular, greater disability after a stroke is associated with neglect, especially after damage to the right hemisphere of the brain (Durfee, & Hillis, 2023). In addition, in approximately 40% of patients, this syndrome persists a year after its onset (Van Kessel et al., 2013). Therefore, finding ways to overcome unilateral neglect remains an important task.

The rehabilitation of patients with neglect syndrome has been predominantly studied by scientists such as Goldstein, Zangwill, Diller, and Luria (1948); Ben-Yishay (1985); Prigatano and Niemeier (1998); and Wilson and Marshall (2009) (Winson, Wilson & Bateman, 2017; Weinberg et al., 1977). These researchers have made significant contributions to the understanding of the problem.

Studies testing rehabilitation methods for patients with neglect syndrome are often criticized for their methodological limitations (Chen, et al, 2022; Checketts et al., 2021; Umeonwuka, Roos, & Ntsiea, 2022; Veldema et al., 2020). Cognitive rehabilitation has the major disadvantage of not being able to transfer trained skills into real life in most cases. Patients demonstrate the effectiveness of performing most tasks under clinical conditions, but struggle to apply the acquired knowledge to everyday situations.

To reduce the gap between function and functioning in patients with neglect syndrome, special rehabilitation training has been developed. This training consists of environmentally friendly tasks and methods specifically designed to overcome the challenges faced by these patients.

Rehabilitation training is understood in neuropsychology as a system of specially designed tasks aimed at overcoming a particular disorder (Yurina, Stepanov, et al., 2023). Its purpose is, first, to make patients aware of their own defect, and 2) then to overcome existing disorders, if possible, primarily by restructuring the functional system and/or functioning, and further adapting patients with cognitive impairments to the conditions of everyday life and adequate functioning in it (Varako et al., 2019).

The founders of cognitive training who focused on the rehabilitation of patients with spatial disorders after a stroke in the right hemisphere were Diller and Ben-Yishay, who, in collaboration with Weinberg (1977), in the 1970s conducted a number of studies at the Institute of Restorative Medicine of New York University.

During the initial rehabilitation training, the patients were put in a room with light bulbs installed on an iron grate. The patients were instructed to observe the lights as they turned on and off sequentially from right to left. This exercise gradually encouraged them to shift their focus towards the left side and expand their visual field to include not only the right but also the left side of the space. Consequently, this training strategy became known as “visual scanning training” (Weinberg et al., 1977).

In 1993, American scientists Calvanio, Levine, and Petrone identified two approaches to cognitive training as a means of rehabilitation: 1) training aimed at overcoming systematic impairment (Impairment Training), and 2) training aimed at overcoming the inability to perform a specific task (Task-Specific Training).

The approach focused on overcoming systematic impairment (Impairment Training) defines its goal as solving the problem of a cognitive dysfunction which is common to many tasks. During the first stage of this training, it is necessary to identify the one or more primary impaired mental functions underlying the inadequate performance of various tasks. Furthermore, a program of classes is developed to improve the patient’s capabilities in tasks with similar structures, as well as in their surrounding social and living conditions. The program aims to overcome common disorders (Calvanio et al., 1993).

Task-Specific Training addresses the problem of improving the inadequate performance of a specific task using various cognitive means. Unlike the previous approach, this training emphasizes only reducing the severity of the underlying defect, not overcoming it. The applicability of this training is limited to a specific task, and doesn’t apply to general environmental conditions (Calvanio et al., 1993).

Our training program corresponds to the Impairment Training approach (training aimed at overcoming a systematic violation), as classified by Calvanio, Levine, and Petrone (1993).

## Methods

### Experimental section

The present study was conducted in the period from September 2019 to April 2023 at the Department of Medical Rehabilitation of patients with impaired central Nervous System function, of the Pirogov National Medical and Surgical Center of the Ministry of Health of the Russian Federation. It consisted of two stages.

### Description of Stage I of the study

***Objective of Stage I of the study***. The aim of Stage I was to develop and validate the author’s “Puzzle” task (Propustina et al., 2022) and evaluate its sensitivity in diagnosing neglect syndrome.

***Participants in stage I of the study***. The empirical part of the first stage of the study took place in the period from September 2019 to May 2021. The first stage involved 83 patients with neurological disorders of various etiologies, with a focus on lesions in the region of the right cerebral hemisphere. The participants were 26 women and 57 men, who ranged in age from 36 to 80 years (median = 60 years). The localization and nature of the pathological focus in the brain of the patients were verified by computed tomography.

The criteria for inclusion in the study were the following:

- The ability to be active with at least one hand;
- The absence of gross disorders in the neurodynamic and regulatory spheres;
- A clear state of consciousness;
- Right-handedness, and the absence of left-handedness and ambidexterity in one’s family history.

### Procedure

The participants were divided into two groups: control and experimental. The experimental group included 45 patients with neglect syndrome or a tendency to left-sided neglect; the control group included 38 patients with right hemisphere lesions without signs of neglect syndrome.

Each participant underwent a preliminary neuropsychological examination using various tests (Taylor’s Figure, Bells Test, TMT, and others), the results of which were used to identify the neglect syndrome. Each subject then performed the author’s “Puzzle” task.

This method featured 18 square cardboard pieces (hereinafter referred to as details), each measuring 6 cm x 6 cm, with a letter on the front side. The participants were assigned two tasks. The first task required the patient to divide the parts into three sections: left, middle, and right, and then place them face down. During the second task, patients were required to construct a phrase using cardboard pieces arranged in three stacks of six cards each. The phrase was read out by a psychologist prior to the task. (Propustina et al., 2022).

### Description of Stage II of the study

***Objective of Stage II of the study.*** The aim of stage II was to develop a specialized rehabilitation program aimed at overcoming the neglect syndrome, and to evaluate its effect on visuospatial search performance when used with patients with this disorder (Yurina et al., 2023).

***Participants in stage II of the study***. The empirical part of stage II took place from September 2021 to April 2023. Thirty-seven patients with neurological disorders of various etiologies with a lesion focused in the right hemisphere of the brain participated in phase II; the group included 8 women and 29 men ranging in age from 36 to 76 years (median = 60 years), The localization and nature of the pathological focal point in the brains of the patients were verified by computed tomography.

### Procedure

All patients during the preliminary neuropsychological examination showed neglect syndrome. The study primarily focused on the manifestations of unilateral neglect in the visual sphere.

The study divided stage II patients into three groups: two control groups and one experimental group. The experimental group consisted of 14 patients who underwent specialized rehabilitation training to overcome neglect syndrome. The first control group consisted of five patients who underwent non-specialized rehabilitation training to overcome social skills impairment. The second control group comprised 18 patients who did not participate in any of the rehabilitation programs.

### Methods of exposure

Specialized training was used to rehabilitate patients in the experimental group and overcome neglect syndrome. The training was designed to be used in both group and individual formats within the clinic (Propustina et al., 2021). The methodological basis for the creation of this work was:

1. P.Y. Galperin’s theory of systematic-stage formation of mental actions and concepts (Talyzina, 1998; Galperin, 2017);
2. The concepts of mediation and arbitrariness according to Vygotsky (1982);
3. The hierarchical structure of rehabilitation and holistic approach of Ben-Yishay (1985);
4. Exercises to train visual attention abilities (Winson et al., 2017).

The training consisted of four meetings with the patients lasting 45 minutes each. The format was individual-group, which ensured the active participation of each and every patient in the sessions.

Anosognosia is a common disorder found in patients with right brain damage. It significantly interferes with the patient’s understanding of his/her disease and motivation for rehabilitation, and slows down the rehabilitation process (Calvanio et al., 1993). To overcome anosognosia, a psychoeducation phase was conducted at the beginning of each rehabilitation session to familiarize the patients with the manifestations of neglect syndrome.

The Lighthouse Strategy, developed by American psychologist Niemeier, is a key rehabilitation strategy used for patients with non-eclecticism. This strategy aims to improve rehabilitation outcomes. During the tasks, patients are asked to imagine themselves as a lighthouse, with their eyes serving as the light. According to Niemeier (1998), the task is to visually illuminate the surroundings from right to left, similar to a lighthouse beam, while using the patient’s shoulders as landmarks.

This strategy at the initial stage of rehabilitation is recommended to be supported by verbal prompts or auxiliary poster pictures. At later stages, patients are encouraged to direct the “ray of light” independently, verbally repeating the Lighthouse Strategy during the lesson. Using the strategy trains patients to perform head/eye/body turns from right to left in situations where they have difficulty detecting stimuli on the left.

The training uses tasks aimed primarily at the visual sphere, since the neglect syndrome in this modality has the strongest effect on human functioning (Bartolomeo, 2021).

The content of the first lesson, in addition to the stage of psychoeducation, included familiarizing the patients with the Lighthouse Strategy: *i.e*., the tasks of visual scanning (naming numbers presented on the screen in turn), and recognizing the contents of the “Plate” picture and the “Bingo” game, which is the model for this strategy. The task of recognizing the contents in the “Plate” picture and the game “Bingo” has a social and household character. In the first task, the patient needs to most accurately describe the dishes depicted on a sheet of paper on the right and left sides in the form of a plate; in the second, the principle of the game involves an active search for the desired object among others in a given visual field. It is worth noting that an important role in the performance of these tasks is played by the factor of external motivation, applied through confrontation between the participants in the training.

The second lesson’s structure included a psychoeducation stage where patients were introduced to the polymodal nature of the neglect syndrome. Training tasks focused on using the Lighthouse Strategy. They included the “Crossword Puzzle” task and the task of collecting words in the opposite direction. The tasks were designed to facilitate eye movement in a Lighthouse Strategy, moving from right to left when searching for cells and letters. For object search tasks, patients were provided with a schema-instruction to locate all necessary items on the table.

The third lesson involved psychoeducation and tasks for correlating two concepts: 1) collecting words in the opposite direction, searching for a route; and 2) a task similar to the “Silent Clock” test. The route search task is an interactive activity during which the participants must imagine themselves in the place of the main character and get from the starting point to the end, taking into account all the difficulties that he may face in real life: *i.e*., orientation at the train station and metro station; familiarization with the information on the ticket; searching for a car; following the signs, and other things. The incentive material for the route search task is presented in PowerPoint format and focuses on the most environmentally friendly situations often encountered in everyday life.

The content of the fourth lesson is aimed at generalizing the previously received theoretical information about the neglect syndrome and performing tasks using the learned Lighthouse Strategy (for example, the tasks “Tic-Tac-Toe” and “Search for Differences” in the PowerPoint format).

It is worth noting that most of the tasks of the rehabilitation training relied on external and internal facilitation (auxiliary) signals as stimuli. External signals are aimed at expanding the area of visual scanning and consist of the constant verbal repetition of the Lighthouse Strategy. Internal signals, on the contrary, are aimed at improving the patient’s ability to detect stimulus information on the left and navigate to it, despite the relatively wide scanning area, and are represented by a red auxiliary line on the left in tasks on paper or screen, the left edge of the table/screen/sheet, the patient’s left hand, or a sound cue signal on the left (Calvanio et al., 1993).

After each of the four sessions, the patients were asked to recall the activities they had completed during the previous session. Additionally, they received feedback on their progress towards rehabilitation goals and objectives, and positive reinforcement in the form of a small reward or sweet prize, subject to physician approval.

The first control group received a non-specialized type of rehabilitation, which focused on overcoming violations of social norms and improving social interaction, rather than countering the neglect syndrome. This group served as a comparison sample.

The training was developed based on the clinical recommendations by Haskins et al. in the Handbook of Cognitive Rehabilitation (2012). It consisted of three 45-minute thematic meetings for a group of two to six patients.

The classes of the non-specialized training had a general structure: the stage of psychoeducation on the topics of “Communication and verbal communication,” “Non-verbal communication,” and “Social context” (Adamyants, 2019); a brief theoretical introduction to the topic of the lesson; moderated discussion; practical development of acquired skills in a small group; and feedback on the results of the lesson.

Patients in the second control group, on the contrary, did not participate in any of the rehabilitation programs presented above. Their stay in the clinic was limited to standard individual classes with a medical psychologist (for example, performing tasks like proofreading tests), a speech therapist, a physical therapy instructor, and other specialists, depending on the patient’s diagnosis.

### Research methods

To assess the impact of the specialized rehabilitation training on the indicators of visuospatial search, the ascertaining and control diagnostics of the patients with neglect syndrome were carried out using the author’s “Puzzle” task (Propustina et al., 2022).

As objective indicators, the number of turns of the head to the left, the overall visual activity of the patient, the effectiveness and time of performing the tasks of the test, and the percentage of omissions of stimuli on the left were evaluated.

Indicators of the patient’s overall visual activity and the effectiveness of the tasks of the “Puzzle” test were measured on a two-point scale (0 and 1, respectively). The general level of visual activity was characterized by the presence of a tendency for the patient to study the surrounding space, and was evaluated not only during the performance of specific tasks, but also in the pauses between them. The patient studied what was around him, and turned his head in different directions, thereby capturing most of the space. The performance indicator reflected the successful or unsuccessful performance by the patient of a particular task of the “Puzzle” task (turned over/ did not turn over all the square details, or composed/did not compose a phrase out of them). Indicators of visuospatial search were measured on ordinal and absolute scales.

## Results

Statistical data processing was carried out applying the IBM SPSS Statistics 23.0 system and included the Spearman’s rank correlation coefficient, Wilcoxon’s nonparametric Test, and one-dimensional analysis of variance with repeated measurements of ANOVARM.

## Results of Stage I of the study

First of all, it was necessary to test the author’s “Puzzle” method and confirm its sensitivity for the diagnosis of patients with neglect syndrome. For this purpose, the analysis of both groups was carried out as follows: the differences in the indicators of the average time for completing each task and the total average time for completing the “Puzzle” method in the two groups (*Table 1*) were explained by the fact that patients with neglect syndrome tend to ignore and not notice details on the left side. Such patients spend more time on the visuospatial search for the target stimulus on the left and mainly concentrate on objects located on the right side or in the central part of the table.

**Table 1:** The average execution time of the author’s “Puzzle” task by patients of the experimental and control groups

	Average unconscious task completion time	Average conscious task completion time	Total average execution time of the task
Experimental group	37 sec	2 min 29 sec	3 min 6 sec
Control group	28 sec	1 min 4 sec	1 min 32 sec

Moreover, prior to taking the author’s “Puzzle” test, all patients underwent a comprehensive neuropsychological examination using Luria’s diagnostic techniques and the Trail Making Test (TMT) and Bells Test (Mancuso et al., 2019).

Using Spearman’s nonparametric rank correlation coefficient, the following statistically significant correlations were obtained (*Table 2*):

**Table 2:** Spearman’s rank correlation coefficients by the TMT, Bells Test, and the author’s “Puzzle” task

	Trail Making Test	Bells Test (R-F)
The “Puzzle” test, the 1st task	.490** <.001	-.299** .006
The “Puzzle” test, the 2nd task	.703** <.001	-.621** <.001

Note. **- p-value is less than .01

- a strong positive correlation (r = 0.703, p <.05) between the time of TMT and the second task of composing phrases in the “Puzzle” game;
- a moderately negative correlation^1^ (r = -0.621, p <.05) between the lateralization index during the Bells Test and the time of completion of the second task for composing phrases in the “Puzzle” game;
- a moderately positive correlation (r = 0.490, р <.05) between the execution time of TMT and the first task of flipping pieces in the “Puzzle” game;
- a weak negative correlation (r = -0.299, р <.05) between the lateralization index of the Bells Test and the time to complete the first piece flip task in the “Puzzle” game.

The results showed that the author’s “Puzzle” method has sufficient construct validity, is appropriate, and, along with the specialized tests Trail Making Test and Bells Test, can be used for diagnosing patients with neglect syndrome (p <.001).

## Results of Phase II of the study

Using ANOVARM, the differences in the objective measures of head rotation to the left, and the percentage of missing stimuli on the left, were analyzed in the patients of the three groups participating in Phase II of the study.

The number of head turns to the left side varied over time between the groups (F = 18.127, p<.05) (*Tables 3–4*).

**Table 3:** Multivariate criteria in ANOVARM analysis (objective indicator: head turns)

Effect	Pillai’s Trace	F	The of error freedom degrees	Statistical significance
Factor A Factor A*group	.311 .516	15.356 18.127	34.000 34.000	<.001 <.001

**Table 4:** Criteria for intergroup effects in ANOVARM analysis (objective indicator: head turns)

	Average square	F	Significance
Free member Group	869.049 2.846	146.796 .491	<.001 .617

For patients who participated in non-specialized training, or did not participate in rehabilitation programs at all, the index of head turns to the left remained practically unchanged (the graph lines are almost parallel). The lack of dynamics in the changes in this indicator for the two control groups can be seen when analyzing the mean, which differed when the control testing was compared to the confirmatory one by only 0.06 times (without rehabilitation), and 0.2 times (non-specialized rehabilitation). The absence of significant differences in indicators in patients who did not undergo any training, suggests test-retest reliability of the “Puzzle” task.

For patients who underwent the specialized rehabilitation training, there was a significant dynamic in the change in the number of head turns to the left side: the index of the average between the two tests increased by more than 3.5 times (1.93 on the ascertaining and 5.5 on the control diagnoses).

**Figure 1:**
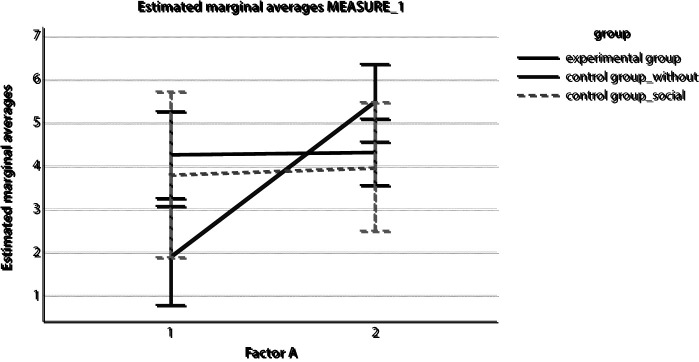
Estimated averages for the indicator “Number of head turns to the left”

*Figure 1* shows that initially, the experimental group was less oriented to the left side of the space than the two control groups. When analyzing the data obtained in the ascertaining diagnosis, it is notable that the patients of the experimental group had a stronger degree of expression of the neglect syndrome; *i.e*., they ignored a greater number of stimuli located on the left side. This disorder in patients of the control groups manifested itself in a mild or moderate degree of neglect.

During the control diagnostics stage, there was no significant difference between the experimental and control groups in the manifestation of the neglect syndrome. However, patients in the experimental group, who initially had a more severe degree of this disorder, showed improvement in the “number of head turns to the left” indicator after participating in specialized rehabilitation training. In contrast, the indicator in the control groups remained the same compared to the confirmatory diagnosis at the beginning of their hospitalization.

Similar data were also obtained using ANOVARM when analyzing the percentage of stimulus misses located on the left side (F = 18.367, p<.05). Patients who had the special rehabilitation training were observed to show a dynamic change in the percentage of left-sided stimulus omissions: the mean between the two slices increased by more than 0.5 times (.68 on the test and .08 on the control diagnoses). The changes in the mean for the two control groups was minimal: –.04 and .001, respectively.

To evaluate the impact of the rehabilitation program on visual search indicators such as total visual activity and task performance, the mean percentages for each group of patients in the determining and controlling diagnostics (percentage of activity and percentage of achievement) were calculated.

*Table 5* shows that positive dynamics in the change of activity and performance indicators were observed only in patients of the experimental group. Patients in the first and second control groups did not show significant improvement in these indicators; they continued to ignore left-sided stimuli, paying attention only to stimuli located on the right side or in the middle of the table.

**Table 5:** Average percentages of the visual search activity and performance in the 2nd task of the “Puzzle” test

	Test		Result	
	Activity	Activity	Retest	Result
Experimental group	21.43%	28.57%	85.71%	85.71%
Control group №1	40%	80%	40%	80%
Control group № 2	50%	77.78%	55.56%	72.22%

The effect of the spatial rehabilitation training on the temporal performance of Puzzle tasks was evaluated using the non-parametric Wilcoxon signed-rank test.

The results showed statistically insignificant differences in the time of performing the test between patients of the experimental group before and after rehabilitation (p>0.05) and patients of the second control group who did not participate in rehabilitation programs (p>.05).

**Table 6:** Assessment of changes in the temporal performance of the “Puzzle” test in patients of the experimental and second control groups

		Wilcoxon T-test	Statistical significance
	Time, 1st task	–1.350	.177
Experimental group	Time, 2nd task	–.220	.826
	Time, 1st task	–.738	.461
Control group №2	Time, 2nd task	–.436	.663

However, upon analyzing the graphs (*Figure 3* and *Figure 4*) obtained using AN-OVARM, the time indices for completing the tasks of the “puzzle” test increased during the confirmatory and control diagnostics. The time taken to complete the author’s test by patients not participating in rehabilitation remained constant upon re-measurement.

During the observation of the experimental group patients’ performance of the Puzzle test, it was noted that the increase in the mean time value was due to an increase in the latency period between the end of the instruction and the beginning of the task (*Table 7*).

**Table 7:** Average time of “Puzzle” test performance by experimental group subjects before and after rehabilitation training

	The ascertaining stage of diagnosis		The control stage of diagnostics	
	Experimental group	Control group No. 2	Experimental group	Control group No. 2
Unconscious task completion time	35 sec	38 sec	41 sec	37 sec
Conscious task completion time	3 min 7 sec	1 min 47 sec	3 min 18 sec	1 min 48 sec

## Discussion

The use of the “Lighthouse” compensatory method increased the patients’ visual search activity and, as a consequence, improved their performance on tasks involving spatially localized stimuli (*e.g.,* card stimuli). Patients in the first and second control groups showed no improvement in these objective measures; they continued to ignore left-sided stimuli and only attended to stimuli located on the right side of the table or in the center.

As a result of participation in the specialized rehabilitation training, patients in the experimental group show significant changes in such objective indicators as head turns to the left and the percentage of stimulus omissions on the left; after participation in training, patients began to pay more attention to the left side of the space, and thus allowed fewer stimulus omissions on the left.

The data obtained during the study practically repeated the results of an experiment conducted by Niemeier, who used the Lighthouse Strategy as a tool in the rehabilitation of patients with visual inattention after a stroke (Niemeier, 1998). In his experiment, patients who participated in comprehensive day rehabilitation programs also increased their ability to attend to the left, and improved their overall attentional skills more than patients who did not use the Lighthouse Strategy.

**Figure 2:**
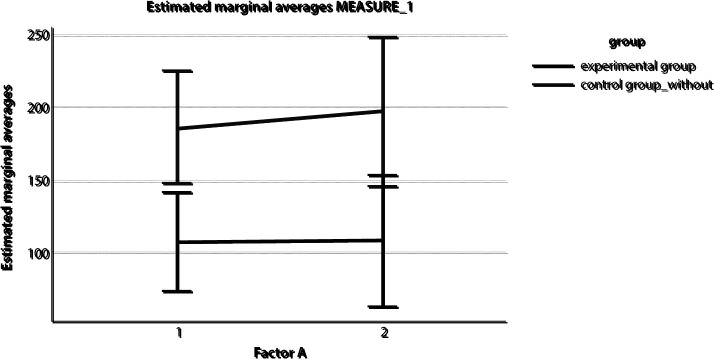
Estimated averages for the indicator “Time to complete the 1st task” with flipping pieces from the “Puzzle” test

Based on the change in objective indicators (FARS Attention’ ratings and the Mesulam Verbal Cancellation Test) before and after rehabilitation lessons, Niemeier concluded that lower functioning patients were actually brought farther along by using the Lighthouse Strategy than patients who were less impacted and exposed to the usual rehabilitation methods of left-sided visual inattention. Similar results were obtained in our study, where patients with a high degree of severity of the syndrome showed a more marked overcoming of left-sided neglect (*Figure 1, Figure 2*).

**Figure 3:**
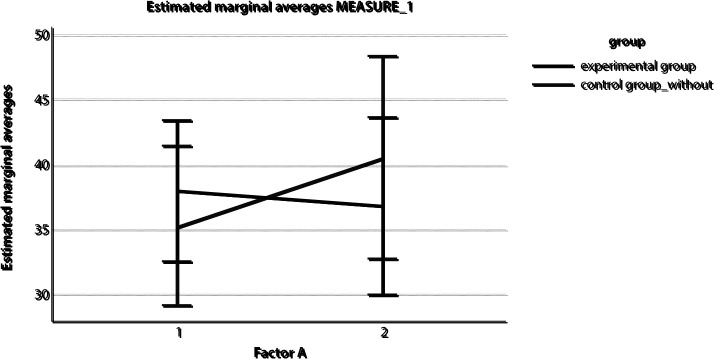
Estimated averages for the indicator “Time of fulfillment of the 2nd phase” collection task from the “Puzzle” test

The results also showed that the time taken to perform the “Puzzle” test increased in the group that received specialized rehabilitation training. It is possible that the patients needed additional time before the task to implement the Lighthouse Strategy. As expected, the visuospatial search in patients with neglect syndrome became more developed due to the newly formed arbitrary, mediated task performance, which is the controlling function of attention.

The Toglia & Chen study (2022) aimed to develop a rehabilitation program for patients with visuospatial neglect. It is noted for its convenience and compactness, consisting of a set of 12-page object search tasks. However, the study presents patients with only flat images, and all tasks are limited to manipulating sheets of paper without extending beyond them. In our study, the application of acquired skills, known as the Lighthouse Strategy, extended beyond the use of sheets of paper. Tasks were displayed on a large screen or around the perimeter of the room. This unique approach is important as it allows patients to simulate real-life situations where they may encounter similar difficulties.

## Conclusion

The uniqueness of the neglect syndrome is determined by its heterogeneous and polymodal nature.

A detailed and accurate study of dissociations in the manifestations of neglect syndrome contributes to the development of more targeted methods of its diagnosis and of rehabilitation programs capable of overcoming or compensating to some extent for the functional deficit caused by this disorder.

The author’s “Puzzle” method was developed and successfully tested in order to diagnose various aspects of the Neglect Syndrome and its connection with the performance of material with different degrees of meaningfulness. Specialized neuropsychological training was developed for the rehabilitation of patients with neglect syndrome.

A comparative analysis of the results of the study before and after training to overcome the neglect syndrome showed significant improvements in the visuospatial search indicators in patients with this disorder. Specialized rehabilitation had a favorable effect on the dynamics of visual search parameters, thus increasing the number of head turns oriented to the left side, improving visual activity and performance, and minimizing the percentage of missed target stimuli localized to the left.

It should be noted that, in addition to the specific effects of the training, the staff of the medical institution also noted non-specific effects, including increased motivation, the tendency for patients to help each other when working in a group, better readiness for individual sessions, and others.

It may be worthwhile to conduct an additional study to evaluate the dynamics of visuospatial search in patients with neglect syndrome using Eye-Tracking technology to objectify eye movement parameters. Additionally, a longitudinal study will be conducted to test whether the skills acquired in using the assistance strategy after a hospital stay are maintained during a re-admission for preventive and rehabilitative measures.

## Limitations

1) The second stage of the study had a small sample size.

The first control group consisted of only five patients with neglect syndrome. The neuropsychological training aimed at overcoming social interaction disorders was created and tested over a period of approximately six months. Due to the limited number of patients with neglect syndrome in the clinic, rehabilitation sessions were only conducted with these five patients during the remaining time. To achieve greater accuracy in the results, it is intended to increase the number of patients in each group during the second stage of the study. This will be accomplished by conducting rehabilitation activities at multiple clinics in Moscow and the surrounding region.

2) Investigation of the impact of specialized rehabilitation training within a single modality.

This study focused solely on the manifestations of neglect syndrome in the visual sphere. This is because these difficulties have the greatest impact on patients’ lives and make them dependent on others. In the future, we plan to use methods to detect left-sided neglect not only in the visual modality but also in the auditory and tactile modalities during the ascertaining and control stages of diagnosis. To achieve this goal, the method of dichotic listening and the Toiber lateralization test of tactile stimuli will be used.

3) Patients have a brief stay in the clinic’s inpatient department.

Patients are admitted to the clinic for a two-week rehabilitation program. During this period, they are required to attend six meetings, including a diagnostic and control stage, as well as four sessions of specialized training. Certain patients were excluded from the final sample of the second stage of the study as they did not participate in all mandatory meetings during the diagnostic and rehabilitation process.

## Footnotes

1 The laterality index is the main diagnostic indicator of Bells Test, reflecting the difference between the number of undetected target stimuli on the right and left sides (Rorden, & Karnath, 2010).
